# A historical review of mycosis fungoides: from Alibert to mogamulizumab

**DOI:** 10.1093/skinhd/vzaf099

**Published:** 2025-12-30

**Authors:** Nicholas A Johnson, Opeoluwa Fariyike, Khaylen Mistry, Zoe Venables, Nick J Levell

**Affiliations:** Norwich Medical School, University of East Anglia, Norwich, UK; Norwich Medical School, University of East Anglia, Norwich, UK; Norwich Medical School, University of East Anglia, Norwich, UK; Department of Dermatology, Norfolk and Norwich University Hospital, Norwich, UK; Norwich Medical School, University of East Anglia, Norwich, UK; Department of Dermatology, Norfolk and Norwich University Hospital, Norwich, UK; Norwich Medical School, University of East Anglia, Norwich, UK; Department of Dermatology, Norfolk and Norwich University Hospital, Norwich, UK

## Abstract

In 1806, French physician Baron Jean-Louis Alibert saw a man with a desquamating rash and skin tumours. Alibert considered this to be a variant of yaws. In 1829 Alibert named the condition mycosis fungoides (MF), meaning ‘mushroom-like fungal disease’. Over 100 years later, French dermatologist Albert Sézary published papers from 1938 to 1949 detailing a mysterious disease containing ‘cellules monstrueuses’, describing cutaneous ‘monster cells’. In 1961, these clinical findings were collated together into ‘Sézary syndrome’. In the 1870s English dermatologist William Tilbury Fox published a dermatology atlas detailing cases similar to what we know now as MF, with the name ‘fibroma fungoides’. The atlas described MF as a type of fungus, before giving a description of yaws and painting a clinical picture that differed from that of a lymphoma. Over the twentieth century, our understandings of the origins of MF were changing and by 1975 the classification system and term we now recognize as cutaneous T-cell lymphoma (CTCL) was developed. Neoplastic cells have been thought to arise from chronic activation of T cells via antigen-presenting cells due to inappropriate cytokines and C-C chemokine receptors. In 2018, the World Health Organization and European Organisation for Research and Treatment of Cancer officially recognized four variants of MF. These are the classic Alibert–Bazin variant and its three variants: folliculotropic MF, pagetoid reticulosis and granulomatous slack skin. Developments in immunohistochemistry for the T-cell receptor gene in the 1990s improved the diagnosis of CTCL; however, diagnosis is still challenging. Advanced MF therapies have evolved from cytotoxic chemotherapy to novel monoclonal antibodies such as mogamulizumab, targeting proteins on T-cell lymphoma cells.

## Teaser

Since the first description of mycosis fungicides in 1806, there has been widespread confusion regarding the nomenclature, aetiology and origin of the disease. This review reflects on the past two centuries, to offer insight into these challenges.

## First understandings of mycosis fungoides, and its clinical diagnosis

**Figure 1:**
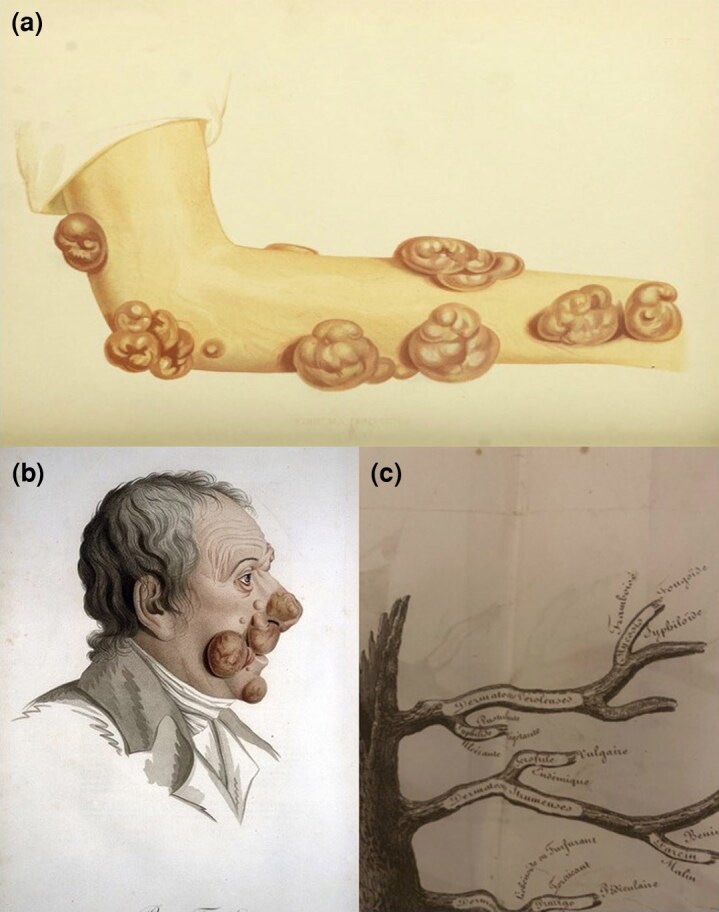
(a) Early illustration of fibroma fungoides taken from Tilbury Fox’s *Atlas of Skin Disease*, 1872, which may represent mycosis fungoides (MF). Credit: Wellcome Library, London, under the Public Domain Universal Licence (https://creativecommons.org/publicdomain/mark/1.0/). (b) Early drawings of MF termed ‘pian fongoide’, taken from skin observed in ‘Description des maladies de la peau observées à l'Hôpital Saint-Louis. Et exposition des meilleures méthodes suivies pour leur traitement / par’, J.L. Alibert 1806. Credit: Wellcome Library, London, under the Creative Commons Attribution Lisence (https://creativecommons.org/licenses/by/4.0/). (c) In J.L. Alibert, Arbre Des Dermatoses in ‘Monographie des Dermatoses’, 1835, MF is grouped with syphilis. Credit: Reproduced from a copy of *Monographie des Dermatoses* gifted to author N.J.L.

Since its first description in 1806 by French dermatologist Baron Jean-Louis Alibert (1768–1837), there has been confusion about the classification and diagnosis of mycosis fungoides (MF). A 56-year-old man named ‘Lucas’ was treated by Alibert for a desquamating rash and skin tumours, which Alibert termed as ‘pian fongoide’.^1^ The term ‘pian’ was used by European physicians to describe yaws (*Treponema pallidum* subspecies *pertenue*),^2^ while ‘fongoide’ described mushroom-like growth. Alibert detailed an ailment that developed small tubercles like a potato or mushroom.^3^ Alibert first used the term ‘mycosis fungoides’ in ‘Monographe des Dermatoses’ in 1835 for the same patient (Figure 1).^4^ This terminology may have caused confusion regarding a possible infective, either fungal or treponemal, aetiology of MF.

In 1852, William James Erasmus Wilson (1809−1884) from England described Alibert’s comments on MF. Wilson disagreed with Alibert, classifying MF with molluscum contagiosum, then known as molluscum of Bateman.^5,6^

In 1862, Alibert’s student, Pierre-Antioine-Ernest Bazin (1807–1878), described three progressive stages of MF: eczematoid, infiltrative and tumoural, which later became known as the ‘Alibert–Bazin’ type.^7^ By 1874, Wilson had given the condition the name ‘eczema tuberculatum’.^8^

In 1877, English dermatologist William Tilbury Fox (1836–1879) published in *Atlas of Skin Diseases* four cases of ‘fibroma fungoides’.^9^ Fox gave the opinion that fibroma fungoides existed separately from lymph adenomas, epitheliomas, contractile keloids and MF of the French, as described by Alibert. A summary of these cases, and their key descriptions, can be found contrasted against Alibert’s ‘pian fongoide’ in Table 1.

**Table 1:** Details and descriptions from Alibert’s and Fox’s reports on patients with mycosis fungoides

Patient number	Date of case	Author	Book	Sex/age (years) if disclosed	Key term(s) to describe lesions	Final diagnosis
Patient 1 (‘Lucas’)	1806	J.L. Alibert	*De la peau observe a L’Hospital St Louis*	Male, 56	Small tubercles	Pian fongoide. Illustration available in Figure 1(b)
Patient 2	1871	W. Tilbury Fox	*Atlas of the Skin* 1875	Male, N/A	Without distinct granulations, fungal ‘excrescences’ Fibrocellular tissue	Fibroma fungoides
Patient 3	Unknown	W. Tilbury Fox	*Atlas of the Skin* 1875	Female, N/A	Fibromatous tumours Fibrocellular tissue	Fibroma fungoides/lymphadenoma/‘mycosis of the French’
Patient 4	Unknown	W. Tilbury Fox	*Atlas of the Skin* 1875	N/A, child	Fibrocellular tissue Fleshy masses resembling half-ripe black grapes	Fibroma fungoides

N/A, not available.

Confusion about the cause and origin of MF was widespread in the early and mid-nineteenth century as diagnosis was by visual examination. In the absence of precise diagnostic criteria and confusing nomenclature, many cases of MF were likely misdiagnosed.

Diagnosis of MF improved in the late nineteenth century with innovation of histopathological techniques. Louis-Antoine Ranvier (1835–1922) demonstrated reticular lymphoid tissues in MF in 1875.^7^ In 1887, French dermatologist Jean Ferdinand Darier (1856−1938) described ‘Darier’s epidermal nests’, later renamed ‘petits nids cellulaires’.^10^

In 1885, Émile Charles Achille Vidal (1825–1893) and Louis-Anne-Jean Brocq (1856–1928) documented MF ‘d’emblee’, in which tumours were the first cutaneous manifestation – instead of being preceded by eruptions or infiltrations.^7^ In 1892, François Henri Hallopeau (1842–1919) and Ernest Henri Besnier (1831–1909) described erythroderma before the appearance of tumours.^7^ Pautrier microabscesses were credited to French dermatologist Lucien Marie Pautrier (1876–1959) in 1927, at the New York Society of Dermatology.^10–12^

French dermatologist Albert Sézary (1880–1956) published papers from 1938 to 1949 detailing a variant of MF with erythroderma and ‘cellules monstrueuses’, or ‘monster cells’, in the blood.^13^ In 1961, these clinical findings were collated together into ‘Sézary syndrome’ (SS) and the cutaneous presence of ‘mononuclear cells’ was identified.^14^

Marvin A. Lutzner (unknown dates) and Richard L. Edelson (1944–) coined the term and classification of cutaneous T-cell lymphoma (CTCL) in 1975, allowing for unity as the term encompassed both MF and SS.^15^ However, MF diagnosis is often still delayed due to similarities in clinical presentation to common conditions such as psoriasis, eczema and tinea infections.^16^

## Advancement and evolution of diagnosing and understanding the aetiology of mycosis fungoides

The distinct yet progressive stages of MF, coupled with limited diagnostic techniques, led to further confusion about its origin. Potential triggers of MF such as environmental and/or occupational exposure to solvents, genetic malformations and infectious causes, such as truncated human T-cell lymphotropic virus (HTLV)-1, have been proposed and rejected.^17–21^

In the mid-twentieth century, the advent of flow cytometry and immunohistochemistry revolutionized MF diagnosis by identifying its T-cell origin and demonstrating antigen markers. In 1968, Lutzner used electron microscopy to comment on the irregularity of SS and MF cell nuclei, and the serpentine nature of SS nuclei, leading to the naming of Lutzner cells in the 1970s.^22,23^ Electron microscopy in 1980 was used to visualize the morphology of viruses such as HTLV, then thought to be involved in MF pathogenesis.^24^ In 1985 the Southern blot test demonstrated the monoclonality of MF cells, and by 1987 immunohistology had helped identify the presence of CD30 antigens on MF cells.^25,26^ The 1990s saw the polymerase chain reaction identify T-cell receptor arrangements specific to MF cells.^27^ However, the laboratory diagnosis of MF remains challenging.^28,29^

In MF, malignant T cells express cutaneous ­lymphocyte-associated antigen (CLA) and C-C chemokine receptors (CCRs). CLA^+^ T cells migrate to the skin through the help of cytokines, from sources such as keratinocytes.^30^ One of the important CCRs seen in MF is CCR4 and its ligand CCL17. They are involved in the apprehension of T cells and increasing prosurvival factors such as phosphatidylinositol-3-­kinase, allowing these T cells to become resistant to apoptosis.^31^ MF cells can lack receptors such as CD7, CD5 and CD2, and malignant T cells congregate around Langerhans cells – seen as Pautrier microabscesses.^30^ Patients with MF have higher serum interleukin (IL)-7 and IL-15, which have roles in memory T-cell proliferation.^32^

In 2018, the World Health Organization and European Organisation for Research and Treatment of Cancer officially recognized four variants of MF (Table 2).^33^

**Table 2:** Clinical variants of mycosis fungoides and their origin

Clinical variants	First clinical description of variant	Origin of term
Alibert–Bazin	1806: Alibert, later commented upon by Bazin	Named from Alibert’s and Bazin’s contributions
Pagetoid reticulosis (Woringer–Kolopp disease)	1939: Frederic Woringer (1903–1964) and Pierre Kolopp^34^	1973: term proposed by Otto Braun-Falco (1922–2018)^34^
Folliculotropic	1960: Hans-Georg Piper (1911–1969)^35^	1985: term ‘follicular’ mycosis fungoides proposed by Sun-Yong Kim^35^
Granulomatous slack skin	1973: Jacinto Convit (1913–2014)^36^	1978: term ‘granulomatous cutis laxa’ proposed by Albert Bernard Ackerman (1936–2008)^36^

## How has mycosis fungoides management evolved?

As MF was confused as a variant of yaws/syphilis in the early nineteenth century, treatments may have included various herbal compounds, arsenic, atoxyl, sodium casodylate, potassium iodide and mercury.^37^

Fox’s nineteenth-century treatments for MF (as fibroma fungoides) included hygienic regimens, nutritious diets and nourishment, unspecified tonics and astringents applied directly to the sores.^9^

The twentieth and twenty-first centuries brought many advancements in the management of MF (Table 3). Many treatments listed in Table 3 are still used in 2025.^68^ Psoralen and ultraviolet A, total skin electron beam therapy and topical nitrogen mustards are still indicated in both early- and advanced-stage management, as monotherapy or in conjunction.^30^

**Table 3:** Treatments for mycosis fungoides: their inception and first description in managing mycosis fungoides (MF)

Treatment	Inception and first clinical use	First use in MF
Radiotherapy	1896: Used to treat malignancy, a year after the discovery of X-rays^38^	1951: TSEBT used in MF^39^ and later in other T-cell lymphomas^40^
NM gas and mechlorethamine	1946: Clinical pharmacologists Louis Goodman and Alfred Gilman used NM-based compounds such as methyl-bis (beta-chloroethyl) amine to manage Hodgkin lymphoma, leukaemia and other malignancies^41^	1959: MF treated with topical mechlorethamine at Cleveland clinic by John R. Haserick and Joseph H. Richardson:^42^ alkylating agent with a strong antiproliferative effect^43^
Photochemotherapy	1948: A.M. el-Moft used purified 8-MOP for vitiligo.^44^ Thomas B. Fitzpatrick researched and developed psoralens,^45^ and by 1974 with John A. Parrish coined the term PUVA^46^	1976: Fitzpatrick treated MF.^47^ In the 1970s and 1980s, trials of other photochemotherapeutic agents.^48–50^ In 1989, a trial ran by Frederic J. Kaye compared radiation therapy and chemotherapy with topical PUVA and found no improved prognosis^51^
Interferons	1957: First named and described.^52^ 1974: Jordan U. Gutterman first clinical trial of IFN-α in cancer^53^	1984: Paul A. Bunn Jr described efficacy of high-dose recombinant IFN-α in patients with CTCL^54^
VAD	1962: Topical tretinoin and other VAD compounds were used in malignancies and hyperkeratotic disorders^55^	1983: John F. Kessler uses isotretinoin in CTCL.^56^ 1999: bexarotene was approved by the FDA^57^
HDACi	1969: Akira Inoue isolated histone deacetylases.^58^ 1998: Victoria M. Richon found SAHA and m-CBHA effective against MEL and cancer cells^59^	2006: Vorinostat (SAHA) was approved by the FDA for CTCL,^60^ followed by romidepsin in 2009^61^
Monoclonal antibodies	1986: The first monoclonal antibody, orthoclone OKT3, was used to reduce rejection in kidney transplantation.^62^ 2012: Mogamulizumab, an anti-CCR4, was first approved in CCR4^+^ adult T-cell leukaemia/lymphoma^63^	2002: Alemtuzumab, an anti-CD52, monoclonal antibody, was first trialled in CTCL.^64^ 2017: Brentuximab vedotin was approved by the FDA for CD30-expressing MF.^65^ 2018: Mogamulizumab was first licensed by the FDA,^66^ and in 2021 by NICE^67^

CBHA, carboxycinnamic acid bishydroxamide; CCR, C-C chemokine receptor; CTCL, cutaneous T-cell lymphoma; FDA, Food and Drug Administration; HDACi, histone deacetylases inhibitors; IFN, interferon; MEL, murine erythroleukaemia; 8-MOP, 8-methoxypsoralen; NICE, National Institute for Health and Care Excellence; NM, nitrogen mustard; PUVA, psoralen and ultraviolet A; SAHA, suberoylanilide hydroxamic acid; TSEBT, total skin electron beam therapy; VAD, vitamin A derivatives.

In recent years monoclonal antibodies such as mogamulizumab have improved progression-free survival rates in MF.^69^ Mogamulizumab targets the CCR4 receptor, which – with its complementary CCL17 ligand – inhibits dermal migration of MF cells.^70^ Treatments for MF continue to improve, and although it is understood that MF is not an infectious disease, infections such as *Staphylococcus aureus* can promote MF.^71^

## Summary

Reflection on the past two centuries from Alibert to mogamulizumab offers insight into the challenges that accompany the aetiology, diagnosis, classification and management of MF. While MF management has improved, diagnosis is often delayed due to clinician and patient confusion caused by the similarities in presentation with common benign dermatoses. Further patient and clinician education must be done to optimize patient outcomes.

## Data availability

No new data were generated in this review.
